# Hexokinase-2-Linked Glycolytic Overload and Unscheduled Glycolysis—Driver of Insulin Resistance and Development of Vascular Complications of Diabetes

**DOI:** 10.3390/ijms23042165

**Published:** 2022-02-16

**Authors:** Naila Rabbani, Mingzhan Xue, Paul J. Thornalley

**Affiliations:** 1 Department of Basic Medical Science, College of Medicine, Qatar University Health, Qatar University, Doha P.O. Box 2713, Qatar; 2 Diabetes Research Center, Qatar Biomedical Research Institute, Hamad Bin Khalifa University, Qatar Foundation, Doha P.O. Box 34110, Qatar; mxue@hbku.edu.qa

**Keywords:** hexokinase-2, hyperglycemia, glycolysis, diabetes, diabetic complications, insulin resistance, methylglyoxal, glyoxalase 1

## Abstract

The recent discovery of the glucose-induced stabilization of hexokinase-2 (HK2) to proteolysis in cell dysfunction in model hyperglycemia has revealed a likely key initiating factor contributing to the development of insulin resistance and vascular complications in diabetes. Consequently, the increased flux of glucose metabolism without a change in the expression and activity of glycolytic enzymes produces a wave of increased glycolytic intermediates driving mitochondrial dysfunction and increased reactive oxygen species (ROS) formation, the activation of hexosamine and protein kinase C pathways, the increased formation of methylglyoxal-producing dicarbonyl stress, and the activation of the unfolded protein response. This is called HK2-linked glycolytic overload and unscheduled glycolysis. The conditions required to sustain this are GLUT1 and/or GLUT3 glucose uptake and the expression of HK2. A metabolic biomarker of its occurrence is the abnormally increased deposition of glycogen, which is produced by metabolic channeling when HK2 becomes detached from mitochondria. These conditions and metabolic consequences are found in the vasculature, kidneys, retina, peripheral nerves, and early-stage embryo development in diabetes and likely sustain the development of diabetic vascular complications and embryopathy. In insulin resistance, HK2-linked unscheduled glycolysis may also be established in skeletal muscle and adipose tissue. This may explain the increased glucose disposal by skeletal uptake in the fasting phase in patients with type 2 diabetes mellitus, compared to healthy controls, and the presence of insulin resistance in patients with type 1 diabetes mellitus. Importantly, glyoxalase 1 inducer—*trans*-resveratrol and hesperetin in combination (tRES-HESP)—corrected HK2-linked glycolytic overload and unscheduled glycolysis and reversed insulin resistance and improved vascular inflammation in overweight and obese subjects in clinical trial. Further studies are now required to evaluate tRES-HESP for the prevention and reversal of early-stage type 2 diabetes and for the treatment of the vascular complications of diabetes.

## 1. Introduction: Human Hexokinase-2—Overview of Molecular Characteristics and Subcellular and Tissue Expression

Hexokinase-2 (HK2; EC:2.7.1.1) is one of four isozymes of hexokinase in mammalian metabolism that catalyze the first step of glucose metabolism, the conversion of glucose to glucose-6-phosphate (G6P): Glucose + MgATP → G6P + MgADP. That is, hexokinases interact with glucose and the magnesium Mg^2+^ complex of ATP [1]. Uniquely, HK2 has a second glucose-binding active site [1]. This additional active site contains a degradation motif that binds heat shock protein cognate 70 (HSC70) and directs HK2 for proteolysis by chaperone-mediated autophagy [2]. Human HK2 is a monomeric protein of molecular mass 100 kDa and K_M_ for glucose of 340 µM [1]. HK2 is normally localized mostly to the external surface of mitochondria [3] and is found in the particulate extracts of tissues [4]. The N-terminal domain of HK2 binds to voltage-dependent anion channel protein (VDAC), located within the outer mitochondrial membrane and acting as a conduit for ATP and for the supply of HK2-catalysed reaction [5,6]. HK2 is the major isozyme of hexokinase in skeletal muscle and adipose tissue. It also has significant expression, along with hexokinase-1 (HK1), in vascular cells, as well as the kidneys, the retina, and Schwann cells in the peripheral nerves; it is also the major hexokinase isozyme expressed in early-stage embryogenesis [7,8]. HK2 has high expression levels in many types of human tumor, together with increased expressions of phosphofructokinase (PFK), other glycolytic enzymes, and glucose-6-phosphate dehydrogenase (G6PD). This supports increased flux through glycolysis for tumor growth without an increase in the intermediates of early-stage glycolysis [9,10]. The promoter of HK2 in tumors is often hypomethylated, which is associated with the dysregulation of transcription—including the direct binding of glucose to increase expression [11,12]. Tumor cell lines may exhibit the direct induction of HK2 expression by glucose, whereas non-malignant human cells in primary culture do not [11,13]. The use of tumor cell lines to study dysfunctional metabolism in model hyperglycemia may therefore give misleading outcomes. The genetic polymorphism of HK2 was studied as a possible factor linked to the risk of developing type 2 diabetes mellitus (T2DM) in the Finnish population, but no significant association was found [14,15]. A novel genetic polymorphism of HK2 was found in Pima Indians—a Native American tribe with a high prevalence of T2DM—but it displayed no significant association with risk of T2DM [16].

## 2. Hexokinase-2-Linked Glycolytic Overload and Unscheduled Glycolysis

In studies of metabolic dysfunction supporting dicarbonyl stress induced by high glucose concentrations in human aortic endothelial cells and human periodontal ligament fibroblasts in primary culture, we discovered that the increased glucose metabolism driving metabolic dysfunction in model hyperglycemia was mediated by glucose-induced stabilization of HK2 to proteolysis, producing HK2-linked glycolytic overload [13,17]. This was corrected by off-target effects of glyoxalase 1 (Glo1) inducer, *trans*-resveratrol, and hesperetin in combination (tRES-HESP) [13,17]. In this review, we describe this and evidence that the hypothesis of HK2-linked glycolytic overload and unscheduled glycolysis are likely key initiators of metabolic dysfunction, contributing to the development of vascular complications of diabetes, diabetic embryopathy, and insulin resistance.

**Figure 1:**
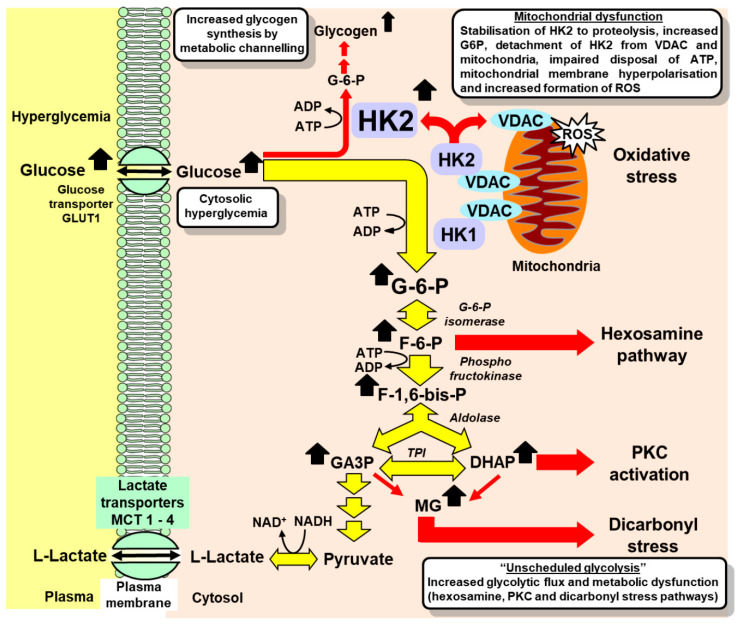
Glycolytic overload and unscheduled glycolysis in hyperglycemia. Key: red arrows—dysfunctional metabolism in unscheduled glycolysis. Metabolic intermediates in glycolysis from GA3P to pyruvate have been omitted for clarity. Abbreviations: DHAP, dihydroxyacetonephosphate; F-6-P, fructose-6-phosphate; F-1,6-bis-P, fructose-1,6-bisphosphate; G-6-P, glucose-6-phosphate; GA3P, glyceraldehyde-3-phosphate; HK1, hexokinase-1; HK2, hexokinase-2; MCT 1–4, monocarboxylate transporters 1–4; MG, methylglyoxal; ROS, reactive oxygen species; VDAC, voltage-dependent anion channel.

During periods of high glucose concentration in the cytosol, the degradation motif of HK2 is masked by increased active site occupancy by glucose. This stabilizes HK2 to proteolysis, increasing HK2 protein abundance and activity [13,17], and increasing flux of glucose metabolism without any change in other glycolytic enzyme activities and expression—generating a wave of increased glycolytic intermediates through early-stage glycolysis. The consequences of this are:

(i) Increased levels of G6P, which displaces HK2 from mitochondria, impairing the disposal of ATP—causing mitochondrial membrane hyperpolarization, mitochondrial dysfunction, and the increased formation of reactive oxygen species (ROS) [3,18,19,20];
(ii) Increased fructose-6-phosphate and activation of the hexosamine pathway, increasing enzymatic protein glycosylation [19,20];
(iii) Increased formation of glycerol-3-phosphate and formation of diacylglycerol, with the consequent activation of protein kinase C (PKC) [19,20];
(iv) Increased glyceraldehyde-3-phosphate (GA3P) and dihydroxyacetonephosphate (DHAP), leading to the increased formation of methylglyoxal and dicarbonyl stress [13,19,20];
(v) Increased metabolic channeling of G6P for glycogen synthesis as a consequence of the displacement of HK2 from mitochondria [3] (Figure 1).

In primary cultures of human aortal endothelial cells and periodontal ligament fibroblasts, the flux of glucose metabolism was increased ca. twofold in model hyperglycemia [13,17]. A similar effect was found previously in bovine aortal endothelial cells in culture [21].

Under normal metabolic regulation and in short periods of increased plasma glucose concentration, the increased flux of glucose metabolism through glycolysis at sites of vascular complications is restricted by saturation of HK1 and HK2. This also applies for extended periods of increased plasma glucose concentration for tissues with predominantly only HK1 expression—such as the brain. When increased flux of glucose metabolism in skeletal muscle and adipose tissue occurs in response to insulin, regulatory activation and/or the increased expression of glycolytic enzymes occurs such that steady-state levels of glycolytic intermediates remain unchanged or are increased modestly [22]. Responses to insulin include: increased activity of glucose transporter GLUT4, increased expression and activity of HK2, increased activity of 6-phosphofructo-2-kinase/fructose-2,6-bisphosphatase (increasing allosteric regulator, fructose-2,6-bisphosphate), and thereby of phosphofructokinase, and increased expression and activity of glyceraldehyde-3-phosphate dehydrogenase (GA3PD) [23,24,25,26]. With the prolonged increase of glucose metabolism and moderate increases in G6P, the nuclear translocation of the Mondo A/Mlx/G6P transcription factor complex increases the expression of a battery of glycolytic and lipogenic genes with a functional carbohydrate response element (ChRE) [27,28,29]. This provides a further level of regulation of gene expression in early-stage glycolysis. The paralog protein, Mondo B, or carbohydrate response element binding protein (ChREBP), is dominant in the liver and adipose tissue [30]. Through this regulatory control, an increase in flux in glucose uptake and metabolism occurs without marked increases in G6P, F6P, or triosephosphates [22], avoiding mitochondrial dysfunction and the activation of hexosamine, PKC, and dicarbonyl stress pathways. This is regulated, “scheduled glycolysis”, and avoids metabolic dysfunction. By contrast, HK2-linked glycolytic overload is unscheduled glycolysis and the multiple pathways of metabolic dysfunction thereby activated are linked to the vascular complications of diabetes, diabetic embryopathy, and ischemia-reperfusion injury, as reviewed previously [31] and presented below for the first time in relation to insulin resistance.

## 3. Hexokinase-2-Linked Glycolytic Overload versus Oxidative Stress as an Initiator of Metabolic Dysfunction in Hyperglycemia

For ca. 20 years, the “*mitochondrial dysfunction-linked oxidative stress in vascular cells with GLUT1-dependent glucose uptake*” hypothesis has been the leading hypothesis proposed to explain the initiation of metabolic dysfunction in hyperglycemia driving the development of vascular complications of diabetes [32]. The increased formation of ROS in vascular cells in hyperglycemia also occurs through the activation of vascular NADPH oxidase (NOX) and the increased expression and uncoupling of endothelial nitric oxide synthase (NOS3), stimulated by upstream activation of PKC [33,34,35]. The hypothesis implicated increased glucose metabolism in hyperglycemia as a driver of mitochondrial and metabolic dysfunction but provided no explanation as to how the saturation of one or both of the two hexokinase isozymes found at the sites of development of vascular complications, HK1 and HK2, is circumvented. A prediction of this hypothesis was that the antioxidant treatment of vascular complications of diabetes would provide effective therapy. However, clinical trials of the antioxidant treatment of the vascular complications of diabetes have been disappointing, showing no or limited benefits. Examples include vitamin E therapy in the Heart Outcomes Prevention Evaluation (HOPE) and Microvascular Outcomes Prevention Evaluation (MICROHOPE) studies [36] and studies of α-lipoic acid in diabetic neuropathy [37]. The hypothesis also provided no explanation for why the peripheral nerves and brain both suffer cytosolic hyperglycemia in diabetes—as indicated by increased N_ε_-fructosyl-lysine content of protein extracts of sciatic nerve and brain in experimental diabetes [38]—but dysfunction and pathogenesis is found predominantly in the neurons of peripheral nerves and not in the brain [39]. The hypothesis of HK2-linked glycolytic overload provides an explanation for all these unresolved features of pathogenic mechanisms and therapy.

In the “*HK2-linked glycolytic overload and unscheduled glycolysis*” hypothesis, the cytoplasmic hyperglycemia-induced stabilization of HK2 to proteolysis provides the initiating mechanism of increased glucose metabolism, circumventing the substrate saturation of hexokinases (Figure 1). Antioxidant therapy may be ineffective because mitochondrial dysfunction and the formation of ROS are not the initiators of metabolic dysfunction but are rather among multiple downstream effects of the initiating process. Finally, HK2 expression occurs in Schwann cells in peripheral neurons but has very low expression in the neurons of the central nervous system (CNS), providing a rationale for why the brain is resistant and the peripheral nervous system is sensitive to hyperglycemia-induced metabolic dysfunction in diabetes [40,41]. Both peripheral and CNS neurons suffer cytosolic hyperglycemia in diabetes, with GLUT1 and GLUT3 glucose uptake, but only Schwann cells have significant expressions of HK2 and are susceptible to HK2-linked glycolytic overload [31]. Impaired functional support of Schwann cells for axons of peripheral neurons may mediate the development of diabetic neuropathy [39,42].

**Figure 2:**
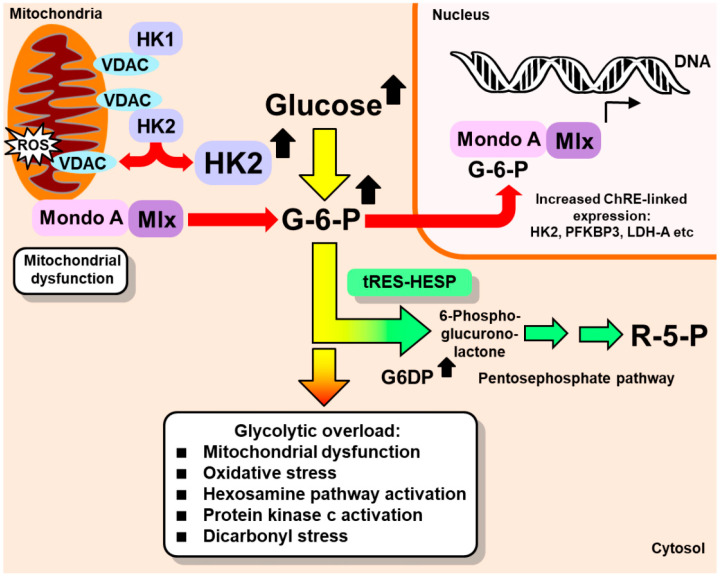
Alleviation of HK-2-linked unscheduled glycolysis by induction of glucose-6-phosphate dehydrogenase expression. Red-tipped arrows: potentially damaging effects; green arrows, Glo1 inducer, tRES-HESP, effect. G6PD, glucose-6-phosphate dehydrogenase; LDH-A, lactic dehydrogenase, isoform A; PFKBP3, 6-phosphofructo-2-kinase/fructose-2,6-bisphosphatase, isoform 3; R5P, ribose-5-phosphate; ROS, reactive oxygen species; VDAC, voltage-dependent anion channel [31].

In the HK2-linked glycolytic overload and unscheduled glycolysis hypothesis, effective therapy can be provided by decreasing HK2 expression to protein abundance and activity levels found in normoglycemia. This was achieved with tRES-HESP—a synergistic combination of two dietary bioactive compounds optimized to induce the expression of Glo1 via activation and the binding of transcription factor Nrf2 to a functional antioxidant response element (ARE) in the GLO1 gene [43,44]. A further Nrf2-mediated effect was induction of the expression of G6PD—also an ARE-linked gene. This decreases cellular G6P, decreasing the Mondo A/Mlx/G6P-dependent expression of HK2, thereby correcting HK2 protein to normal levels in high glucose concentration (Figure 2). Under treatment with tRES-HESP, human aortal endothelial cells and periodontal ligament fibroblasts were cultured in high glucose concentrations with minimal metabolic dysfunction [13,17]. This offers a new approach for the prevention and treatment of insulin resistance, the vascular complications of diabetes, and diabetic embryopathy.

## 4. Evidence of Hexokinase-2 Linked Glycolytic Overload Occurring at Sites of Vascular Complications of Diabetes

An inspection of previous studies reveals widespread evidence for HK2-linked glycolytic overload involvement in the development of endothelial dysfunction in diabetes, and vascular complications of diabetes —diabetic nephropathy, diabetic retinopathy and diabetic neuropathy, and diabetic embryopathy (Table 1). The increased basal expression of HK2 in dermal fibroblasts cultured from patients with type 1 diabetes mellitus (T1DM) was also linked to the rapid progression of diabetic nephropathy, suggesting that basal HK2 expression may be a risk predictor of the progression of vascular complications of diabetes [45]. The constituent cell types exhibiting the criteria for susceptibility to HK2-linked glycolytic overload are: vascular endothelial cells; mesangial cells, podocytes, and tubular epithelial cells of the kidney; endothelial cells, Muller cells, and pericytes of the retina; and Schwann cells of the peripheral nervous system [13,46,47,48,49]. Early-stage embryonic cells—up to day 10 post-conception for mouse embryos—have mainly glucose uptake by GLUT1 and GLUT3 and HK2-linked glycolysis [7,50]. From days 2 to 10 post-conception of embryo development, glucose metabolism is mainly anaerobic glycolysis—predisposing embryo development to dysregulation in the early-stage glycolysis during this period [51]. All the features of HK2-linked glycolytic overload have been reported for early-stage embryos in high-glucose concentration cultures and experimental diabetic embryopathy [7,52,53,54,55]. There is evidence, therefore, that HK2-linked glycolytic overload occurs at the sites of vascular complications and early-stage embryo in diabetes and this likely contributes to hyperglycemia-linked pathogenesis developing therein in diabetes. Similar pathogenesis, of lower severity, is expected in prediabetes, consistent with the low risk and presence of vascular complications and embryo malformations in prediabetes [56,57].

Increased deposition of glycogen was a surprising mechanistic biomarker of HK2-linked glycolytic overload [13]. Increased glycogen deposition was found previously in experimental diabetes in renal proximal and distal tubules, linked to diabetic kidney disease, and in the retina, linked to diabetic retinopathy [84,85]. It was also found in Schwann cells in experimental and clinical diabetic neuropathy and linked to severity of nerve damage [86,87]. Increased glycogen deposition was also found in corneal neurons in diabetes [88] and in early-stage embryos incubated in high glucose concentrations [83]. Glycogen deposition at the sites of vascular complication in diabetes and early-stage embryogenesis in hyperglycemia linked to pathogenesis has hitherto been an inexplicable phenomenon; now, however, it is a prediction of, and consistent with, the HK2-linked glycolytic overload hypothesis.

## 5. Evidence for Hexokinase-2 Linked Unscheduled Glycolysis in Insulin Resistance and the Development of Type 2 Diabetes

Insulin resistance in skeletal muscle is considered to be the primary initiating metabolic defect driving the development of T2DM; it is often present many years before diabetes develops [89,90]. Pancreatic beta-cells respond with compensatory increased secretion of insulin, producing hyperinsulinemia. This stresses beta-cell metabolism, insulin production, and glucose homeostasis. Eventually, the hypersecretion of insulin leads to beta-cell failure, with the development of persistent hyperglycemia and the onset of T2DM. Insulin resistance is defined as “*a reduced response of target* *tissues (to insulin),* *compared with subjects with normal* *glucose tolerance*” [91]. The main target tissues of insulin are skeletal muscle, liver tissues, and adipocytes. Skeletal muscle is the predominant site of insulin-mediated glucose uptake in the postprandial state. Hence, the dysfunction of glucose uptake and metabolism by skeletal muscle has a major impact on glucose homeostasis [92].

In human skeletal muscle, HK2 is the major hexokinase isozyme—accounting for ca. 60% of hexokinase activity in the soluble fraction and 90% hexokinase activity in the particulate fraction bound to mitochondria. Insulin increases the expression of HK2 in skeletal muscle [93] and the binding of HK2 to mitochondria, likely by increasing phosphorylation by protein kinase B—also called Akt [94,95]. HK2 mRNA, protein and activity were decreased in patients with T2DM [96], presumably as a consequence of decreased insulin responsiveness and regulation of HK2 expression. We note that to establish unscheduled glycolysis, it is not necessary for the absolute metabolic flux through glycolysis to be relatively high—which occurs in naïve muscle with insulin treatment [97]. Rather, the critical feature is increased glucose uptake by GLUT1 and metabolism, and glucose-linked proteolysis stabilization of HK2 occurring without other regulatory signaling—such as likely occurs when fasting plasma glucose (FPG) is increased.

Given the expression of GLUT1 in human skeletal muscle [98,99], muscle may be susceptible to HK2-linked unscheduled glycolysis when FPG is increased; this can thereby initiate metabolic dysfunction, leading to insulin resistance. Supporting evidence is described below.

*a*. *Overexpression of GLUT1 in skeletal-muscle-induced impairment of insulin-responsive glucose uptake*

Studies of transgenic mice with overexpression of GLUT1 in skeletal muscle showed there was a 3–4 fold increase in basal glucose uptake and no stimulation of glucose uptake by insulin in skeletal muscle, relative to wild-type control mice, whereas wild-type controls had a 2–3 fold increase in glucose uptake in response to insulin [100]. The investigators, Mueckler et al., commented: “*Although blood glucose levels are reduced and fuel metabolism in general is altered in the transgenic animals, we could detect no detrimental effect of these changes on the mice*” [100]. On further examination, the same group characterized the mechanism of impaired insulin stimulation of skeletal muscle in GLUT1-overexpressing mice: there was impaired recruitment of GLUT4 for glucose transport by insulin [101]. They explored the mechanism and found that it involved increased hexosamine pathway activity [102]. Increased hexosamine pathway activity is an expected consequence of HK2-linked unscheduled glycolysis [31]. The impaired recruitment of GLUT4 for increased glucose transport in response to insulin is one of the characteristics of clinical insulin resistance [103]. By contrast, the overexpression of GLUT4 enhanced insulin sensitivity [104].

The overexpression of GLUT1 in skeletal muscle is expected to increase the in situ rate of uptake of glucose in the fasting state, mimicking the effect of increased FPG with normal GLUT1 expression. This suggests that increased glucose uptake in the fasting phase, in the absence of increased insulin and recruitment of GLUT4 glucose transporters, may drive the development of impaired GLUT4 responsiveness to insulin—conditions sustaining HK2-linked unscheduled glycolysis.

There is evidence of the decreased expression of GLUT1 in the skeletal muscle of patients with T2DM, but the effect of increased FPG more than compensates for this to produce an increased rate of whole body glucose utilization in the fasting state [99]. It is, perhaps, hitherto surprising that patients with T2DM and insulin resistance may exhibit increased glucose metabolism in skeletal muscle in the fasting state, compared to healthy controls. HK2-linked unscheduled glycolysis explains this.

*b*. *Partial knockdown of hexokinase-2 (HK2 (−/+)) in mice improved glucose tolerance in the late stage of glucose challenge*

In mice with heterozygous deletion of HK2, there were indications of improved glucose tolerance in the late stage of intraperitoneal glucose load. HK2(+/−) mice had lower plasma glucose and insulin concentrations at 60 min after glucose challenge [105]. The benefits of decreased HK2 on the glucose tolerance test are expected to develop at the late stage of the glucose challenge because the stabilization of HK2 by glucose produces a time-dependent increase in HK2 protein abundance linked to metabolic dysfunction. This surprising finding was presented without explanation and requires further investigation. Lower basal levels of HK2 may provide resistance to unscheduled glycolysis. The heterozygous knockdown of HK2 can be viewed as a genetic control to counter the approximate doubling of HK2 in high-glucose concentration-induced unscheduled glycolysis [13,17].

*c*. *Overexpression of hexokinase-2 in skeletal muscle of mice impaired uptake of glucose on a high fat diet in hyperinsulinemic euglycemic clamp studies*

Transgenic mice overexpressing HK2 in their skeletal muscle were generated [106]. Subsequent studies with standard and high-fat diet (HFD) feeding [107] showed that fasting blood glucose was increased in HFD compared with standard diet-fed wild-type mice but not in transgenic HK2-overexpressing mice. In hyperinsulinemic euglycemic clamp studies, the index of glucose uptake, R_g_, was reported for gastrocnemius, superficial vastus lateralis and soleus muscle during saline and insulin infusions. There was no difference in R_g_ when saline alone was infused, whereas it was increased during insulin infusion in all muscles regardless of genotype or diet. For insulin-stimulated glucose uptake, the overexpression of HK2 increased R_g_ in all muscles, compared to wild-type controls, of standard-diet-fed mice. The authors report that HFD feeding “*blunted insulin-stimulated R_g_*” and “*HK2* *overexpression* *was unable to correct the impaired response*”. Whilst this is correct, in the HK2 transgenic mice, there was a ca. 60–70% decrease in R_g_ (*p* < 0.05) with HFD feeding, compared to standard-diet feeding, whereas in the wild-type mice, there was a small, ca. 10–30%, decrease in R_g_ with HFD feeding, compared to standard diet feeding with no significance reported—Figure 4 of Ref. [107]. This indicates that HK2 overexpression impaired insulin-stimulated glucose uptake. It may be that the authors did not describe this as it was an unexpected effect, counter to their hypothesis that “*impaired* *muscle glucose uptake* *resulting from high-fat feeding would be exposed in high* *flux* *states and could be corrected by HK2* *overexpression*” [107]. The concentration of G6P in skeletal muscle was increased in the sedentary state with overexpression of HK2 in the HFD-fed mice [107]. This is consistent with the occurrence of HK2-linked unscheduled glycolysis in the fasting state and the impairment of insulin-stimulated glucose uptake by muscle measured in clamp studies. Increased HK2 expression in the normal diet may improve clamp glucose uptake through high basal levels of glucose phosphorylation together with insulin and other regulatory transcriptional control of glycolytic enzymes.

A further relevant study focused on the skeletal muscle glucose uptake and metabolism in mice with overexpression of both GLUT1 and HK2 in their skeletal muscle. These mice had 3.2 fold increased G6P concentrations and 7.5 fold increased glycogen levels in their skeletal muscle on a standard diet. There was no change in basal or insulin-stimulated whole-body glucose disposal [108]. In this transgenic model, skeletal muscle is chronically exposed to increased G6P, which is expected to induce the increased transcription of glycolytic enzymes through Mondo A/Mlx/G6P signaling. This negates the unscheduled glycolysis that occurs when HK2 abundance occurs without an increase in the transcription and activity of other glycolytic enzymes. In HK2-overexpressing mice (with a 7 fold increase in skeletal muscle HK2 protein [108]), it is expected that there will be a marked increase of HK2 in the cytosol detached from the mitochondria. This non-mitochondrial HK2 increases metabolic channeling for glycogen synthesis in both the fasting and prandial phases [3,13]. This may explain the observed increased glycogen deposition in skeletal muscle in this transgenic model.

*d*. *Downstream metabolic signaling in skeletal muscle and adipose tissue in insulin resistance resembles HK2-linked unscheduled glycolysis*

The metabolic dysfunction characteristic of HK2-linked unscheduled glycolysis is found in insulin resistance in skeletal muscle: hyperpolarization of mitochondria and increased ROS formation, increased hexosamine pathway activity, and activation of PKC and dicarbonyl stress [58,59,60,61]. Similar effects have been recorded in adipose tissue—apart from the activation of the hexosamine pathway [63,64,65,66,67]. For the abnormal deposition of glycogen as a biomarker of HK2-linked unscheduled glycolysis, this is confounded by the decreased insulin signaling in insulin resistance in skeletal muscle and the dysregulation of insulin-stimulated glycogen deposition [91,109]. However, increased glycogen deposition was found in adipose tissue in an animal model of insulin resistance and in clinical obesity [67,110] (Table 1). 

Currently, insulin resistance has no well-evidenced and accepted causative mechanism and has been called a “*malady without a mechanism*” [103]. HK2-linked unscheduled glycolysis may provide a missing contributory mechanism. The current hypotheses of the mechanism of insulin resistance do not explain points a–c above. It has been proposed that glucose entering muscle cells via GLUT4 is only available for glycolysis and glycogen synthesis, whereas glucose entering by GLUT1 is available for other processes contributing to insulin resistance, such as the hexosamine pathway [111]. However, the glucose released from GLUT1 and GLUT4 transporters inside muscle cells is a common glucose pool. The HK2 unscheduled glycolysis hypothesis explains these observations through the entry of glucose via GLUT1 independent of insulin, leading to increased HK2 through stabilization to proteolysis and a wave of increased glycolytic intermediates. By contrast, entry of glucose via GLUT4 stimulated by insulin, with a concomitant stimulation of HK2 expression, phosphofructokinase activity, and the expression of other glycolytic and glycogenic enzymes, produces increased flux through glycolysis and glycogen synthesis without marked increases in steady-state glycolytic intermediates—as discussed in [31]. The stabilization of HK2 to proteolysis with increased glucose metabolism and a related increase in G6P and Mondo A/Mlx/G6P signaling may play a key role in the nutrient sensing of skeletal muscle in fasting and insulin-resistant states. Indeed, Mondo A is also known to be a contributary factor to the development of insulin resistance through its increasing of the expression of thioredoxin interacting protein (TXNIP) [112], impairment of insulin signaling, and increasing of lipogenesis [113,114].

From the hypothesis of HK2-linked unscheduled glycolysis in the development of insulin resistance, two key predictions follow.

**Prediction** **1.** *Increased fasting plasma glucose concentration is an expected risk predictor for the development of insulin resistance and T2DM.*

There is, indeed, clinical evidence to support this. Increase in FPGs measured approximately 3 years apart was a risk predictor of development of increased insulin resistance and T2DM, independent of change in 2 h plasma glucose in an oral glucose tolerance test [115]. FPG was also linked to both hepatic and extrahepatic insulin resistance [116].

**Prediction** **2.** *Insulin resistance is an expected characteristic of subjects with increased fasting plasma glucose, including patients with T1DM.*

The increased FPG found in patients with T1DM is expected to induce insulin resistance. Indeed, studies by DeFronzo et al. found that insulin resistance was a prominent feature of patients with T1DM, linked mainly to insulin resistance in peripheral tissues, particularly skeletal muscle [92,117]. This was independent of changes in the insulin-sensitizing hormone, adiponectin [118]. Hitherto, no explanation has been proposed for this.

Further studies are now required to explore evidence for this hypothesis. A related new approach to the treatment of insulin resistance is tRES-HESP, which corrects HK2-linked unscheduled glycolysis [13,17]. In overweight and obese subjects in the Healthy Aging Through Functional Food (HATFF) clinical trial [44], this treatment corrected insulin resistance and therefore offers a new route to the prevention, reversal, and treatment of T2DM.

## 6. How May Glyoxalase 1 Inducer, *trans*-Resveratrol, and Hesperetin Prevent the Development of Vascular Complications of Diabetes and Correct Insulin Resistance in Skeletal Muscle and Adipose Tissue?

tRES-HESP was developed through the optimization of the induction of Glo1 expression using a screen of dietary bioactive compounds and synergistic combinations, thereby activating Nrf2 and increasing Glo1 transcription by binding to a functional ARE of the GLO1 gene [43,44]. Increased concentrations of MG and MG-mediated protein glycation produce increased misfolding of proteins and activation of the unfolded protein response (UPR) [13,17,119]. Protein glycation by MG is particularly damaging because it often occurs in the functional sites of proteins, producing misfolding and inactivation. It also targets chaperonin complexes and proteasome subunits, which are required for correct protein folding and removal of damaged proteins, respectively. That is, protein glycation by MG also impairs the activities of proteins that correct misfolding or target degradation-misfolded proteins [13]. Further evidence of the impact of MG on protein misfolding includes the increased Golgi-to-endoplasmic reticulum retrograde transport and ubiquitin E3 ligases involved in misfolded protein degradation in fibroblasts under dicarbonyl stress in high glucose concentrations [17], likely supporting endoplasmic reticulum-associated protein degradation of misfolded proteins in the UPR [119]. The prevention of protein glycation by MG by increasing the expression of Glo1 is a therapeutic strategy to prevent and slow the development of the vascular complications of diabetes, as reviewed in [120]. However, in correcting HK2-linked glycolytic overload, tRES-HESP may have a more profound therapeutic response by normalizing multiple pathways of metabolic dysfunction in high glucose concentration by addressing the initiating source of biochemical dysfunction (Figure 1 and Figure 2). For example, with tRES-HESP treatment, human aortal endothelial cells maintained normal levels of glucose metabolism when cultured in high glucose concentrations [13]. No other treatment previously proposed for the vascular complications of diabetes produced this. This new approach to therapy may be more effective because it addresses the likely initiating mechanism of the hyperglycemia-linked mechanisms contributing to the development of the vascular complications of diabetes.

From the hypothesized contribution of HK2-linked unscheduled glycolysis to the development of insulin resistance, the activation of the UPR provides a key contributory role, increasing the expression of established mediators of insulin resistance, TXNIP and tumor necrosis factor-α (TNFα). In UPR activation, inositol requiring enzyme-1α (IRE1α) stabilizes TXNIP mRNA to increase its expression and activity [121]. TXNIP decreases glucose uptake by GLUT4 in skeletal muscle, pancreatic beta-cell mass, and insulin secretion, and increases hepatic gluconeogenesis [122,123,124]. Inflammatory signaling mediated through X box-binding protein 1 (XBP1) of the UPR increases histone H3 lysine 4 methyltransferase, SET7/9, increasing the expression of p65 in the NF-κB system and inflammatory mediators [125,126], including TNFα—a key contributor to insulin resistance in skeletal muscle [127,128]. The correction of insulin resistance by tRES-HESP in overweight and obese subjects in the HATFF study correlated with improvements in the expression of TXNIP and TNFα [129].

tRES-HESP also increased the expression of G6PD, decreasing cellular levels of G6P, thereby decreasing the transcriptional action of Mondo A/Mlx/G6P and the expression of HK2 [13]. If HK2-linked unscheduled glycolysis contributes to insulin resistance, decreases in HK2 expression are expected to decrease insulin resistance and improve glucose tolerance. Decreases in Mondo A/Mlx/G6P transcriptional activity are also expected to decrease the expression of other ChRE-linked genes—TXNIP, lipogenic enzymes, and others (Figure 2)—which may improve glucose tolerance and decrease insulin resistance [113,130]. Interestingly, functional genomic studies with tissue-selective activation of Nrf2 (by partial knockdown of Keap1) in the obesogenic HFD-fed mouse model of insulin resistance indicated that the selective activation of Nrf2 in skeletal muscle and the liver corrected insulin resistance and dysglycemia, respectively [131]. There is the expectation, therefore, that the activation of Nrf2 by tRES-HESP will also produce both of these responses, which was found in the HATFF study [44].

**Figure 3:**
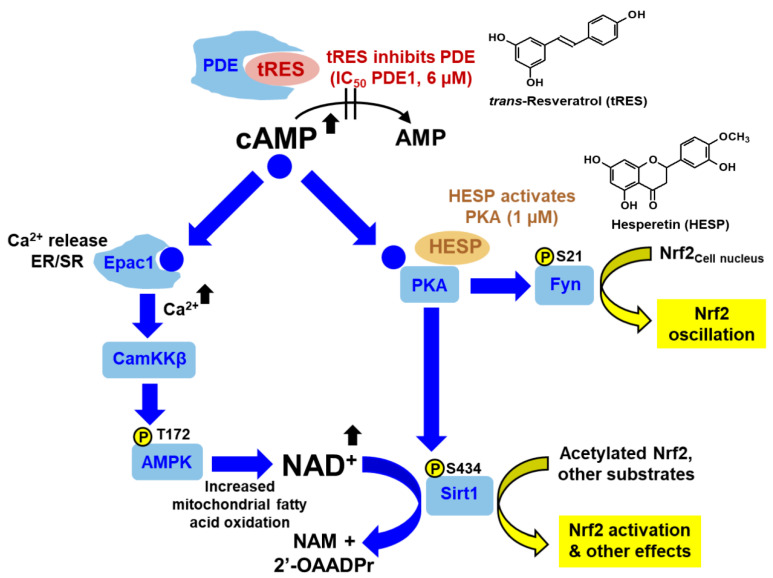
Putative upstream mechanism for activation of transcription factor Nrf2 by tRES-HESP. Abbreviations: AMPK; AMP-dependent kinase; CamKKß, calcium/calmodulin-dependent protein kinase kinase-beta; Epac1, exchange protein directly activated by cAMP 1; ER, endoplasmic reticulum; Fyn, NAM, nicotinamide; 2′-OAADPr, 2′-*O*-acetyl-ADP-ribose; PDE, cAMP phosphodiesterase; PKA, protein kinase A; sirt1; sirtuin-1; and SR, sarcoplasmic reticulum.

We have proposed that tRES and HESP synergize to activate Nrf2 through the upstream inhibition of phosphodiesterase (PDE) [132] and the activation of protein kinase A (PKA) [133], respectively (Figure 3). The Nrf2 system is a constitutive translocational oscillator, with oscillations of Nrf2 moving in and out of the cell nucleus, increasing in frequency when activated [134]. HESP likely drives increased Nrf2 oscillation frequency through the activation of PKA and Fyn kinase downstream at ≥1 µM [44,133,135], and tRES decreases the acetylation-driven nuclear inactivation of Nrf2 by increasing in situ activity by inhibiting cAMP phosphodiesterases, activating AMP-activated protein kinase (AMPK), and increasing NAD^+^ and the in situ activity of sirtuin-1 [132], with HESP also synergizing for AMPK activation and sirtuin-1 through the PKA pathway [132,133,136].

There are multiple pharmacological synergisms of tRES and HESP, including the activation of sirtuin-1 (Sirt1) by both increased provision of NAD^+^ cofactor and activatory phosphorylation by PKA. The activation of Sirt1 and AMPK may also contribute to the correction of insulin resistance by Glo1 inducer. The activation of AMPK is considered to mediate the health-beneficial effects of exercise and caloric restriction, and to contribute to the mechanism of action of metformin—a widely used drug in the treatment of T2DM [137,138]. Metformin is not a competent activator of Nrf2 clinically: the peak plasma concentration of metformin is ca. 20 µM, whereas the activation of Nrf2 by metformin occurs in the concentration range of 600–2700 µM [139,140]. Metformin improves dysglycemia in patients with T2DM mainly by decreasing hepatic glucose production [141]. This is likely through a PKA-dependent mechanism [142], as well as through some improvement in the insulin sensitivity of peripheral tissues by mechanisms that remain unclear [143]. It is interesting to speculate that, through the improvement of dysglycemia, metformin may decrease HK2-linked unscheduled glycolysis and thereby contribute to partial improvement in insulin resistance and the prevention of the vascular complications of diabetes. As an indication of its ability to decrease metabolic dysfunction and its likely activation of the UPR, metformin decreased plasma MG concentration in patients with T2DM [144].

## 7. Concluding Remarks

HK2-linked glycolytic overload and unscheduled glycolysis offers an improved hypothesis to explain metabolic dysfunction contributing to the development of insulin resistance and the vascular complications of diabetes. A dietary supplement treatment, tRES-HESP, has already been developed and evaluated to exploit this hypothesis with beneficial effects in overweight and obese subjects in correcting insulin resistance and improving dysglycemia and vascular inflammation [44,129]. Further studies are now required to evaluate tRES-HESP for the prevention and early-stage reversal of T2DM and the treatment of the vascular complications of diabetes.

## Figures and Tables

**Table 1:** Evidence for hexokinase-driven glycolytic overload and unscheduled glycolysis in insulin resistance, vascular complications of diabetes, and diabetic embryopathy.

Pathogenesis	Tissue/Cell Type	Indications	References
Insulin resistance (skeletal muscle)	Skeletal muscle myocytes	HK2 expression. Downstream metabolic dysfunction (DS, HP, MD, OS, PKC)	[58,59,60,61,62]
Insulin resistance (adipose tissue)	Adipose tissue, insulin-resistant 3T3-L1 adipocytes in vitro	HK2 expression Increased glycogen deposition in adipose tissue Downstream metabolic dysfunction (DS, MD, OS, PKC)	[63,64,65,66,67]
Diabetic endothelial dysfunction	Endothelial cells	Increased glucose metabolism in hyperglycemia through stabilization of HK2 to proteolysis Glycogen accumulation induced by high glucose concentration in vitro and hyperglycemia in vivo Downstream metabolic dysfunction (DS, HP, MD, OS, PKC)	[13,19,20,68]
Diabetic nephropathy	Renal mesangial, cells, podocytes, and tubular epithelial cells	Increased HK2 protein in human mesangial cell by high glucose concentration in vitro Abnormal glycogen deposition in proximal and renal tubules Downstream metabolic dysfunction (DS, HP, MD, OS, PKC)	[46,69,70,71,72,73]
Diabetic neuropathy	Schwann cells (also dorsal root ganglia and sciatic nerve)	Increased HK2 in hyperglycemia Glycogen accumulation in association with demyelination and axonal degeneration in clinical diabetic neuropathy Downstream metabolic dysfunction (DS, MD, OS)	[41,74,75,76,77,78]
Diabetic retinopathy	Muller cells, endothelial cells and pericytes (also intact retina)	HK2 expression in human retina Abnormal glycogen accumulation Downstream metabolic dysfunction (DS, HP, MD, OS, PKC)	[47,73,79,80,81,82]
Diabetic embryopathy	Early-stage embryo (typically rat embryo, day 9–11 gestation)	HK2-dependent glucose metabolism. Increased embryo glycogen content after culture in high glucose concentration in vitro Downstream metabolic dysfunction (DS, HP, MD, OS, PKC)	[7,52,53,54,55,83]

Abbreviations: DS, dicarbonyl stress; HP, hexosamine pathway; MD, mitochondrial dysfunction; OS, oxidative stress; PKC, protein kinase C pathway.

## Data Availability

Data sharing is not applicable to this article.

## Abbreviations

AMPK, AMP-activated protein kinase; ARE, antioxidant response element; ChRE, carbohydrate response element; ChREBP, carbohydrate response element binding protein; CNS, central nervous system; DHAP, dihydroxyacetonephosphate; DS, dicarbonyl stress; F6P, fructose-6-phosphate; FPG, fasting plasma glucose; GA3P, glyceraldehyde-3-phosphate; GA3PD, glyceraldehyde-3-phosphate dehydrogenase; Glo1, glyoxalase 1; G6P, glucose-6-phosphate; G6PD, glucose-6-phosphate dehydrogenase; HFD, high fat diet; HK1, hexokinase-1; HK2, hexokinase-2; HP, hexosamine pathway; HSC70, heat shock protein cognate 70; IRE1α, inositol requiring enzyme-1α; MCT 1–4, monocarboxylate transporters 1–4; MD, mitochondrial dysfunction; NOS3, endothelial nitric oxide synthase; NOX, NADPH oxidase; OS, oxidative stress; PDE, phosphodiesterase; PDLF, periodontal ligament fibroblasts; PFK, phosphofructokinase; PKA, protein kinase A; PKC, protein kinase C; T1DM, type 1 diabetes mellitus; T2DM, type 2 diabetes mellitus; tRES-HESP, *trans*-resveratrol and hesperetin combination; ROS, reactive oxygen species; SET7/9, histone H3 lysine 4 methyltransferase; Sirt1, sirtuin-1; TXNIP, thioredoxin interacting protein; UPR, unfolded protein response; VDAC, voltage dependent anion channel protein; XBP1, X box-binding protein 1.
